# Histopathology of gastrointestinal neuroendocrine neoplasms

**DOI:** 10.3389/fonc.2013.00002

**Published:** 2013-01-22

**Authors:** Kenichi Hirabayashi, Giuseppe Zamboni, Takayuki Nishi, Akira Tanaka, Hiroshi Kajiwara, Naoya Nakamura

**Affiliations:** ^1^Department of Pathology, Ospedale Sacro Cuore Don CalabriaNegrar, Verona, Italy; ^2^Department of Pathology, Tokai University School of MedicineIsehara, Kanagawa, Japan; ^3^Department of Pathology, University of VeronaVerona, Italy; ^4^Department of Surgery, Tokai University School of MedicineIsehara, Kanagawa, Japan

**Keywords:** neuroendocrine neoplasm, carcinoid, neuroendocrine carcinoma, gastrointestinal tract, neuroendocrine marker

## Abstract

Gastrointestinal neuroendocrine neoplasms (GI-NENs) arise from neuroendocrine cells distributed mainly in the mucosa and submucosa of the gastrointestinal tract. In 2010, the World Health Organization (WHO) classification of NENs of the digestive system was changed, categorizing these tumors as grade 1 neuroendocrine tumor (NET), grade-2NET, neuroendocrine carcinoma (large- or small-cell type), or mixed adenoneuroendocrine carcinoma (MANEC). Such a classification is based on the Ki-67 index and mitotic count in histological material. For the accurate pathological diagnosis and grading of NENs, it is important to clearly recognize the characteristic histological features of GI-NENs and to understand the correct method of counting Ki-67 and mitoses. In this review, we focus on the histopathological features of GI-NENs, particularly regarding biopsy and cytological diagnoses, neuroendocrine markers, genetic and molecular features, and the evaluation of the Ki-67 index and mitotic count. In addition, we will address the histological features of GI-NEN in specific organs.

## Introduction

Gastrointestinal neuroendocrine neoplasms (GI-NENs) arise from neuroendocrine cells distributed mainly in the mucosa and submucosa of the gastrointestinal tract. Although multiple diagnostic tools, such as computed tomography, magnetic resonance imaging, ultrasonography, serological tests, and endoscopy, have been developed, pathological investigation is needed for diagnosis of GI-NENs. In 2010, the World Health Organization (WHO) announced a new classification system for NENs of the digestive tract to categorize these tumors as neuroendocrine tumor (NET) Grade (G) 1 (G1-NET), G2-NET, neuroendocrine carcinoma (NEC) large- or small-cell type, and mixed adenoneuroendocrine carcinoma (MANEC) (Bosman et al., 2010). This classification is made using Ki-67 index and mitotic count in histological material. Here, we mainly discuss the histopathological features of GI-NEN, including grading and staging, biopsy and cytological diagnoses, neuroendocrine markers, genetic and molecular features, and the evaluation of the Ki-67 index and mitotic count. In addition, we address the histological features of GI-NEN in specific organs.

## General histopathological features of GI-NENs

**Figure 1:**
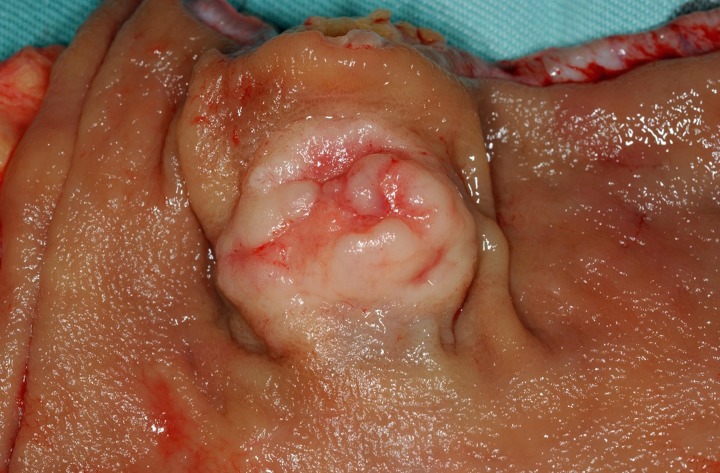
**Rectal neuroendocrine tumor (NET): a solitary sessile mass with slight ulceration**.

**Figure 2:**
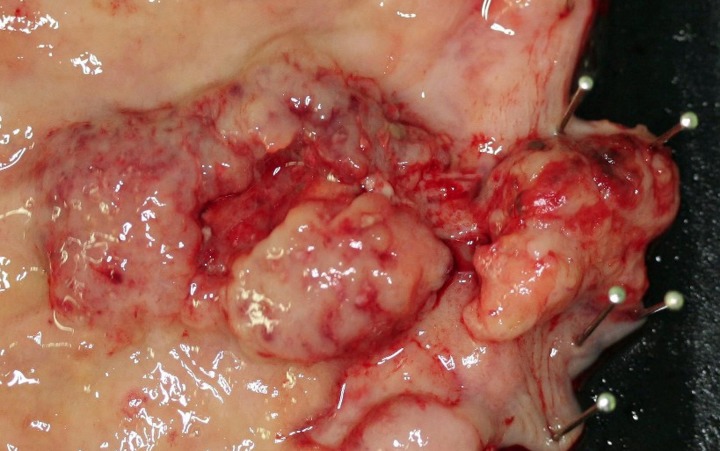
**Neuroendocrine carcinoma (NEC) at the esophagogastric junction: a large ulcerated mass**.

Although the histological features of each GI-NEN depend on the anatomical site and endocrine cell origin, characteristic histopathological features of GI-NENs are generally held in common. Macroscopically, NETs are whitish to yellowish or grayish solid tumors with a nodular or polypoid appearance (Figure 1). The overlying mucosa is generally intact or shows slight focal ulceration because the main lesions of G1- and G2-NETs are deep in the mucosa and submucosa. In contrast, NECs (G3-NETs) are generally larger and occasionally show ulcerated masses as in conventional carcinomas (Figure 2). Microscopically, G1- and G2-NETs are composed of tumor cells possessing round or oval nuclei with “salt and pepper” chromatin and eosinophilic granular cytoplasm. The tumor nests are arranged in trabecular, insular, or sheet-like patterns. NECs are classified as small- or large-cell carcinomas (Bosman et al., 2010). The basic histological features of small- and large-cell carcinomas of the gastrointestinal tract are similar to those of the lung or other organs (Kajiwara et al., 2009; Bosman et al., 2010). Small-cell carcinomas consist of small, round, ovoid or spindle-shaped tumor cells with scant cytoplasm; they are mainly arranged in a sheet-like pattern. Nuclei of small-cell carcinomas show fine granular chromatin with absent or inconspicuous nucleoli. Large-cell carcinomas are composed of medium- or large-sized tumor cells possessing large atypical nuclei with evident nucleoli. The nuclear to cytoplasmic ratio of large-cell carcinomas is lower than that of small-cell carcinomas (Kajiwara et al., 2009; Stojsic et al., 2010). MANECs are malignant tumors composed of both adenocarcinoma and neuroendocrine carcinoma components, with each component accounting for more than 30% of the tumor (Bosman et al., 2010). Squamous cell carcinomas coexisting with NECs have also been reported in the esophagus and duodenum (Yamamoto et al., 2003; Nassar et al., 2005).

## Grading and staging of GI-NENs

In 2010, the WHO launched a new classification system for NENs of the digestive tract, which categorizes them as follows: NET G1, NET G2, NEC (large- or small-cell type), and MANEC (Bosman et al., 2010). NET can be equated with carcinoid (Bosman et al., 2010). “G3-NET” has been used as the category for NEC but is not advised, since NETs are by definition well-differentiated (Bosman et al., 2010). This current WHO classification classifies NENs based only on the Ki-67 index and the evaluation of mitoses in histological material. This classification system is based on the grading system formerly proposed by the European Neuroendocrine Tumor Society (ENETS) (Rindi et al., 2006, 2007). According to the current WHO and ENETS grading systems, G1-NET is designated by a mitotic count of <2 per 2 mm^2^ (10 high power fields [HPF], 40× magnification) and/or ≤2% Ki-67 index; G2-NET by a mitotic count of 2–20 per 2 mm^2^ and/or 3–20% Ki-67 index; G3-NET by mitotic count of >20 per 2 mm^2^ and/or >20% Ki-67 index (Rindi et al., 2006, 2007; Bosman et al., 2010). The survival analysis for foregut NENs (gastric, duodenal, or pancreatic), according to the ENETS-WHO 2010 grading system, showed that survival for patients who had G3 tumors was significantly poorer than that for patients who had G1 and G2 tumors (G1 vs. G3 and G2 vs. G3, *P* < 0.01). Similarly, survival for patients who had G2 tumors was significantly poorer than that for patients who had G1 tumors (G1 vs. G2, *P* = 0.04) (Pape et al., 2008). La Rosa et al. proposed a new global histologic grading system that combined the histologic patterns, based on the WHO 2000 classification, and the ENETS-WHO 2010 proliferative grading system. This global grading system improved tumor prognostic stratification (*P* < 0.001; global grade 1 vs. global grade 2, *P* = 0.007; global grade 1 vs. global grade 3, *P* < 0.001; global grade 2 vs. global grade 3, *P* = 0.001) (La Rosa et al., 2011). The WHO classification requires scanning of at least 50 fields (at 40× magnification) in the areas with the highest mitotic density for the evaluation of the mitotic index in 10 HPF, while ENETS requires at least 40 fields (Rindi et al., 2006, 2007; Bosman et al., 2010). According to the ENETS grading system, 10 HPF corresponds to 2 mm^2^ (Rindi et al., 2006, 2007). However, the size of the HPF differs according to the field number of the eyepiece of each microscope. In breast carcinoma, the adjustment criteria for mitotic count according to the field number of each microscope eyepiece have been proposed (Tsuda et al., 2000). To our knowledge, adjustment criteria for mitotic count according to eyepiece field number for NENs have not been proposed. Accurate grading of NENs might require the development of adjustment criteria for determining mitotic count. The Ki-67 index is calculated as the percentage of Ki-67–positive tumor cells in the areas of the highest density of Ki-67–positive cells, otherwise known as “hot spots.” To evaluate the Ki-67 index, the WHO classification requires 500–2000 tumor cells, while ENETS requires 2000 tumor cells (Rindi et al., 2006, 2007; Bosman et al., 2010). Careful selection of hot spots is crucial for accurate evaluation of the Ki-67 index. In some cases, Ki-67 staining on different and multiple slices could be useful for accurate Ki-67 index evaluation.

So far, two different TNM classifications have been proposed by ENETS and the Union for International Cancer Control (UICC) (Rindi et al., 2006, 2007; Sobin et al., 2009). There are some differences between these staging systems. ENETS staging system applies to all grades of NENs (Rindi et al., 2006, 2007). In contrast, in the seventh edition of the UICC staging system, GI-carcinoid (ENTES G1 and G2) has a specific staging depending on the site of origin, whereas large-cell and small-cell carcinoma (GI-NEC) and all pancreatic NENs are staged like conventional carcinoma (Sobin et al., 2009). Consequently, in the case of pancreatic NENs, a discrepancy in the T-stage (primary tumor stage) between ENETS and UICC staging systems has been observed in 18% cases (Liszka et al., 2011). In the case of GI-NENs, the definitions of T-stage for appendiceal and gastric NENs differ between the two staging systems (see Tables 1 and 2) (Rindi et al., 2006, 2007; Sobin et al., 2009). These differences between ENETS and UICC staging systems may cause confusion in practice and research. Therefore, it is critical to clarify which classification system is being used or document the pathological features, such as tumor size and invasion, that allow for the translation of T-stage between ENETS and UICC classification (Rindi et al., 2006; Kloppel et al., 2010).

**Table 1:** **Comparison of T stage of gastric NENs between ENETS and UICC**.

	**ENETS**	**UICC**
Tis	*In situ* tumor/dysplasia (<0.5 mm)	Mucosa 0.5 mm
T1	Tumor invades lamina propria or submucosa and ≤1 cm	Mucosa 0.5–1 cm or submucosa ≤1 cm
T2	Tumor invades muscularis propria or subserosa or >1 cm	Muscularis propria or >1 cm
T3	Tumor penetrates serosa	Subserosa
T4	Tumor invades adjacent structures	Perforates serosa; adjacent structures

**Table 2:** **Comparison of T stage of appendiceal NENs between ENETS and UICC**.

	**ENETS**	**UICC**
T1	Tumor ≤1 cm invading submucosa and muscularis propria	≤2 cm (T1a, ≤1 cm; T1b, >1–2 cm)
T2	Tumor ≤2 cm invading submucosa, muscularis propria and/or minimally (up to 3 mm) invading subserosa/mesoappendix	>2–4 cm; cecum
T3	Tumor >2 cm and/or extensive (more than 3 mm) invasion of subserosa/mesoappendix	>4 cm; ileum
T4	Tumor invades peritoneum/other organs	Perforates peritoneum; other organs or structures

## Immunohistochemistry of neuroendocrine markers

The histological diagnosis of NENs is generally confirmed by immunohistochemical demonstration of neuroendocrine markers (Hirabayashi et al., 2006; Kajiwara et al., 2009; Yazawa et al., 2011). Several general neuroendocrine markers are known: chromogranin, synaptophysin, protein cell product 9.5, neural cell adhesion molecule (NCAM/CD56), neuron-specific enolase, and Leu 7. Chromogranin A and synaptophysin are the most common markers to confirm the endocrine nature of the neoplastic cells. Chromogranin A is one of the acidic proteins belonging to the chromogranin/secretogranin family and is present in the secretory granules of neuroendocrine cells (Lloyd, 2003). Chromogranin A is an important marker for NENs; however, its expression is focal or negative in NENs with few secretory granules (Wilson and Lloyd, 1984; Lloyd, 2003). Expression of chromogranin A in NETs tends to be strong and diffusely distributed; NECs, in contrast, express chromogranin A weakly or not at all (Rindi et al., 2000b, 2006). In addition, chromogranin A expression tends to be weaker in hindgut NENs (Al-Khafaji et al., 1998). Hindgut NENs are often positive for chromogranin B; therefore, using antibodies against chromogranin B or a combination of staining for chromogranin A and B may be useful for the diagnosis for hindgut NENs (Lloyd, 2003). Synaptophysin is a component of the presynaptic vesicle membrane and is widely distributed in neuroendocrine cells and neurons (Gould et al., 1986; Lloyd, 2003). Unlike chromogranin A, synaptophysin is well preserved in high-grade NENs (Al-Khafaji et al., 1998; Rindi et al., 2006).

## Biopsy and cytology of GI-NENs

**Figure 3:**
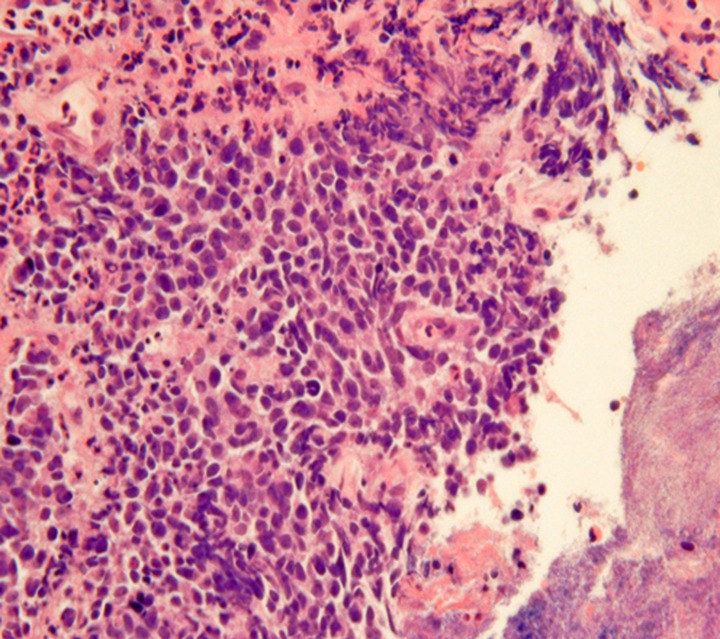
**“Crush”artifacts of esophageal small cell carcinoma (upper right) [hematoxylinand eosin (H and E) stain]**.

Obtaining GI-NEN tissues by endoscopic forceps biopsy is often difficult due to their location in the deep mucosa and submucosa. Even if biopsy is successful, the diagnosis of GI-NEN using biopsy material is sometimes difficult due to small specimen size or “crush” artifacts (Figure 3), which can lead to misdiagnosis (Hoda and Hajdu, 1992; Brenner et al., 2004). Nevertheless, accurate diagnosis from the initial biopsy is important because therapy could differ depending on the diagnosis. Therefore, analysis using neuroendocrine markers should be performed to rule out NENs when tumors have strong crush artifacts in biopsy materials. Fine-needle aspiration cytology is useful for diagnosing GI-NENs because they are generally located in deep areas of the gastrointestinal tract (Benya et al., 1993; Acs et al., 2000; Tasso et al., 2012). Brushing cytology for esophageal small-cell carcinoma also has been reported (Chen, 2000).

Although biopsy and cytology are useful for the diagnosis of NETs, the accuracy of diagnosis and the evaluation of grading using this method can be limited by intratumoral heterogeneity (Couvelard et al., 2009; Yang et al., 2011). Couverlard et al. studied the heterogeneity of the Ki-67 index by comparing two random cores of liver metastasis from pancreatic NENs. They found a good correlation of the Ki-67 index (intraclass correlation coefficient, 0.63) between cores (Couvelard et al., 2009). In contrast, Yang et al. reported that about half of the NENs metastasizing to the liver show intratumoral heterogeneity for Ki-67 grading (G1 vs. G2) on whole-slide subsections (Yang et al., 2011). Furthermore, if biopsy or cytology samples are small, they might not contain adequate numbers of tumor cells for the determination of the Ki-67 index and mitotic count. Another problem is the distribution of neuroendocrine components in MANEC or NENs coexisting with other tumors. Biopsy or cytological materials of MANEC or NENs coexisting with other tumors may not contain neuroendocrine components. This situation can lead to misdiagnosis or difficulty in the diagnosis of NENs on biopsy and cytology. Therefore, in some cases, multiple biopsies or cytological analyses may be needed for accurate diagnosis.

## Genetic and molecular aspects of GI-NENs

Alterations of MEN1 gene (11q13) cause multiple endocrine neoplasia type 1 (MEN1) syndrome. Several sporadic GI-NENs show alterations of MEN1 gene. The incidence of loss of heterozygosity (LOH) of the MEN1gene is 39% of GI-NENs (Rindi and Bordi, 2005). The mutation and promoter methylation of the MEN1 gene are 27% (Rindi and Bordi, 2005) and 23% (Arnold et al., 2007), respectively.

LOH has been occasionally found in the following tumor suppressor genes or chromosomes encoding tumor suppressor genes: chromosome 17p encoding the TP53 gene, chromosome 3p encoding the RAS association domain family 1A (RASSF1A) gene, chromosome 9p encoding p16-INK4a and p14-ARF genes, and chromosome 18q encoding DCC, DPC4/SMAD4, and SMAD2 genes (Terris et al., 1998; Zhao et al., 2000; Lollgen et al., 2001; Chan et al., 2003; Pizzi et al., 2003; Stancu et al., 2003; Pizzi et al., 2005; Rindi and Bordi, 2005). The incidence of TP53 mutation is 4–44% (Ramnani et al., 1999; Rindi and Bordi, 2005). No mutation of the p16-INK4a gene or smad4/DPC4 gene has been reported in GI-NENs (Lollgen et al., 2001; Chan et al., 2003; Stancu et al., 2003).

GI-NENs show frequent methylation of RASSF1A, p14-ARF, retinoic acid receptor beta 2 (RARβ), O6-methyl-guanine methyltransferase (MGMT), cyclooxygenase 2 (COX-2), thrombospondin 1 (THBS1), estrogen receptor (ER), and hypermethylated in cancer 1 (HIC-1) (Chan et al., 2003; Liu et al., 2005; Pizzi et al., 2005; Zhang et al., 2006; Arnold et al., 2007). p16-INK4 methylation is present in 33% of GI-NENs according to the results of several studies (Serrano et al., 2000; Chan et al., 2003; Liu et al., 2005). However, the results of a large series by Arnold et al. show no methylation of p16-INK4 in GI-NENs (Arnold et al., 2007). The methylation of RASSF1A gene is associated with distant or lymph node metastasis (Liu et al., 2005; Zhang et al., 2006).

Several authors have studied the alteration of tumor oncogenes such as HER2/neu, Ras family, and BRAF in GI-NENs. Azzoni et al. reported that 24 cases of ileal NENs showed no amplification of HER2/neu (Azzoni et al., 2011). In contrast, Evers et al. reported that 3 out of 9 cases of GI-NENs showed HER2/neu amplification, and they found a trend of increased HER-2/neu copy number in the more aggressive GI-NENs (Evers et al., 1992). The incidence of immunohistochemical expression of HER2/neu has a broad range (Wang et al., 1997; Yamaguchi et al., 2007; Arnason et al., 2011a; Azzoni et al., 2011). Mutation of the K-ras and BRAF genesis rare in GI-NENs (Younes et al., 1997; Ramnani et al., 1999; Stancu et al., 2003; Perren et al., 2004; Wang et al., 2005; Van Eeden et al., 2007).

Alterations of Wnt signal-associated genes such as beta-catenin and adenomatous polyposis coli (APC) have been reported. Fujimori et al. reported mutations in exon 3 of beta-catenin in 38% (27/72 cases) of GI-NENs and 1 mutation in APC (1%, 1/72 case) (Fujimori et al., 2001). Furthermore, these authors also revealed that 57 out of 72 cases (79%) involve cytoplasmic and/or nuclear accumulation of beta-catenin (Fujimori et al., 2001). However, other authors reported no mutations in the exon 3 beta-catenin or APC gene (Semba et al., 2000; Chan et al., 2003; Stancu et al., 2003; Su et al., 2006), but the incidence of cytoplasmic accumulation and/or nuclear translocation of beta-catenin in GI-NENs was found to be 30–36% (Semba et al., 2000; Su et al., 2006).

Microsatellite instability (MSI) causes several GI carcinomas. The loss of expression of mismatch repair (MMR) proteins relates to MSI. Most MMR proteins (MLH1, MSH2, MSH6, and PMS2) are intact in small intestinal NENs (Kidd et al., 2005; Arnason et al., 2011b) and in colorectal small cell carcinomas (hMLH1, hMSH2, and hMSH6) (Stelow et al., 2006).

As for other molecular alterations, cDNA microarray analysis of GI-NENs reveals that extracellular matrix protein 1 (ECM1), vesicular monoaminemember1 (VMAT1), galectin 4 (LGALS4), and RET proto-oncogene (RET) are highly up-regulated genes (Duerr et al., 2008).

## Characteristics in each specific organ

### Esophagus

**Figure 4:**
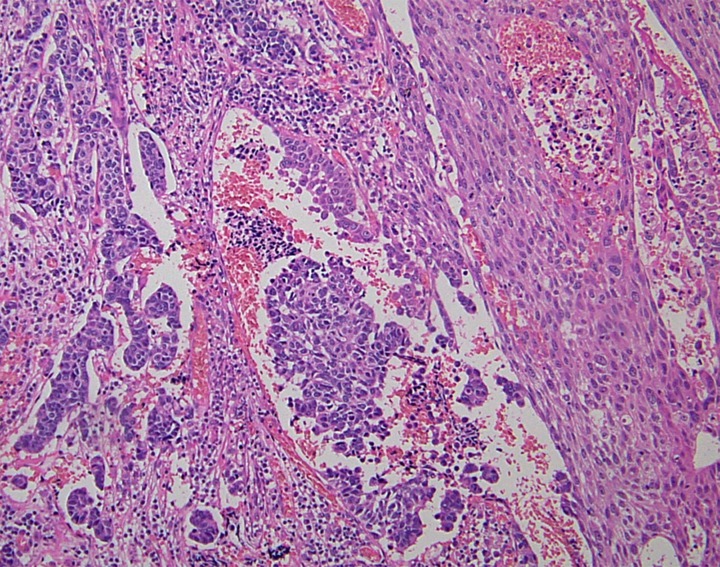
**Combined tumors of neuroendocrine carcinoma (NEC) (left) and squamous cell carcinoma (right) (H and E stain)**.

Most of the NENs arising from the esophagus are NECs, and NETs are rare (Hoang et al., 2002). Esophageal NENs commonly occur in the mid to lower segments of the esophagus (Hoang et al., 2002; Yun et al., 2007). Esophageal NETs appear to arise in two ways: as an incidental finding in association with Barrett esophagus and adenocarcinoma or as a single large polypoid or nodular tumor (Hoang et al., 2002). Upon immunohistochemistry, esophageal NETs are observed to strongly express chromogranin and synaptophysin, and some additionally express serotonin, glucagon, or pancreatic polypeptide (Hoang et al., 2002). Esophageal small-cell carcinoma has similar histological features to lung small-cell carcinoma (Yun et al., 2007). The majority of pulmonary small-cell carcinomas express thyroid transcription factor-1 (TTF-1) (Ordonez, 2000). Similarly, esophageal small-cell carcinomas also often express TTF-1 (33–71%) (Yamamoto et al., 2003; Yun et al., 2007). Combined tumors of NEC and squamous cell carcinoma have been reported (Figure 4) (Yamamoto et al., 2003). Esophageal NECs coexisting with Barrett mucosa or Barrett's esophageal adenocarcinoma have been also reported, as have NETs (Bibeau et al., 2008). Some cases of esophageal NENs show endocrine cell hyperplasia in the Barrett mucosa, as well as invasive adenocarcinoma (Hoang et al., 2002). The presence of endocrine cell hyperplasia in Barrett mucosa and adenocarcinomas supports the hypothesis that esophageal NENs, Barrett mucosa, and adenocarcinomas arise from a common stem cell (Hoang et al., 2002).

### Stomach

Gastric NETs are classified into three types (Rindi et al., 1993; Kloppel et al., 2004, 2007; Scherubl et al., 2010). Type-1 NET is the most common (70–80%). Type-1 NET is related to chronic atrophic gastritis (Rindi et al., 1993; Scherubl et al., 2010) and occur in the corpus and/or fundus of the stomach as multifocal, small, polypoid tumors (<10 mm) (Rindi et al., 1993; Scherubl et al., 2010). Type-2 NETs account for 5–6% of gastric NETs and most commonly arise in the corpus and/or fundus of the stomach as multiple mucosal or submucosal small tumors (<10 mm) (Rindi et al., 1993; Scherubl et al., 2010). Type-2 NET is involved in MEN-1 and Zollinger–Ellison syndrome (ZES) (Rindi et al., 1993; Scherubl et al., 2010). Type-3 NETs account for 14–25% of gastric NETs and occur in any part of the stomach as solitary polypoid tumors (Rindi et al., 1993; Scherubl et al., 2010). These tumors are larger than Type-1 and Type-2 NETs. In one-third of the cases, the tumor was already larger than 2 cm at the time of diagnosis (Kloppel et al., 2004).

**Figure 5:**
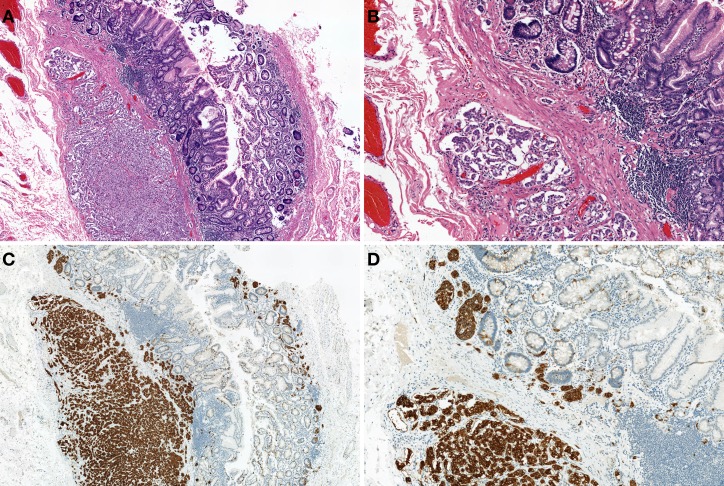
**Type-1 neuroendocrine neoplasm (NEN) of the stomach.** Tumor cells proliferate in a trabecular fashion in the submucosa. Enterochromaffin-like-cell (ECL-cell) hyperplasia is scattered in the mucosa (H and E stain, **A**: low power view, **B**: high power view). Synaptophysin is positive for tumor and ECL-cell hyperplasia (**C**: low power view, **D**: high power view).

Type-1 and Type-2 NETs are associated with enterochromaffin-like-cell (ECL-cell) hyperplasia and hypergastrinemia. In contrast, Type-3 NETs are not associated with these conditions (Scherubl et al., 2010). Type-1, 2, and most Type-3 NETs are considered to originate from histamine-producing ECL-cells which immunohistochemically express vesicular monoamine transporter 2 (Rindi et al., 2000a; Kloppel et al., 2004, 2007; Scherubl et al., 2010). Histologically, Type-1 and Type-2 NETs show a trabecular or nodular pattern (Figures 5A–D). The Ki-67 index is usually less than 2% (Scherubl et al., 2010). Most Type-3 NETs are more aggressive than Type-1 and Type-2 NETs; they are arranged in a solid, trabecular pattern and occasionally have a high proliferation rate (Kloppel et al., 2007; Scherubl et al., 2010). Type-3 NETs often invade deeply, display lymphatic and vascular invasion, and are associated with local and/or distant metastases (Rindi et al., 1999).

NECs present as solitary, large, ulcerated tumors arising in any part of the stomach (Kloppel et al., 2007; Scherubl et al., 2010). Histological features of gastric NECs are similar to those of small-cell or large-cell NETs of the lung (Scherubl et al., 2010).

### Duodenum and proximal jejunum

**Figure 6:**
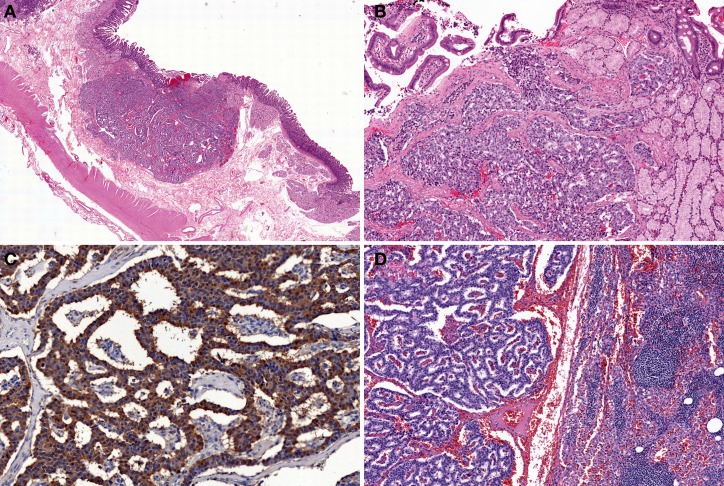
**Gastrin-producing NETs of the duodenum.** Tumor shows trabecular and pseudoglandular patterns (H and E stain, **A**: low power view, **B**: high power view) with immunohistochemical gastrin expression **(C)**. Gastrin-producing NETs metastasize to the lymph node **(D)**.

**Figure 7:**
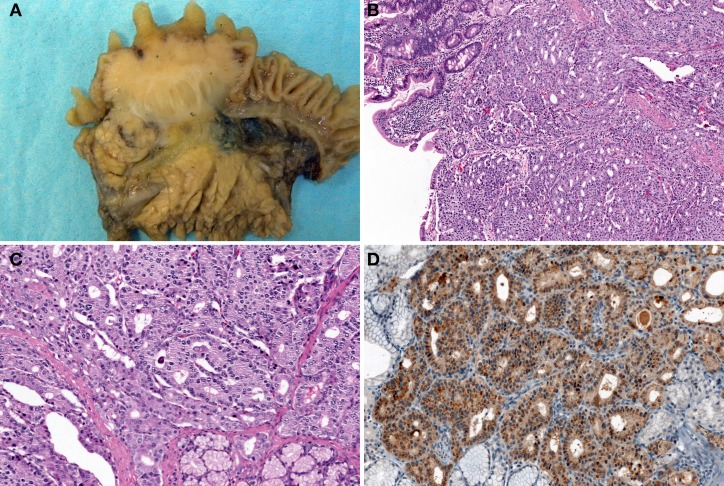
**Somatostatin-producing NETs arising from the papilla of Vater.** Macroscopic findings show the solid, polypoid mass in the papilla of Vater **(A)**. The tumor is arranged in a trabecular pattern with pseudoglandular structures (H and E stain, **B**: low power view, **C**: high power view). Psammoma bodies are present **(C)**. Immunohistochemically, the tumor cells are positive for somatostatin **(D)**.

The most common non-MEN1-associated duodenal NET is gastrin-producing NET (46%), followed by somatostatin-producing NET (18%) (Kloppel et al., 2007; Garbrecht et al., 2008). MEN1 syndrome is associated with 25–33% of duodenal gastrin-producing NETs (Kloppel et al., 2007). Of the gastrin-producing NETs, 47% show functional activity, which causes ZES (Kloppel et al., 2007; Garbrecht et al., 2008). A gastrin-producing NET presents as a small polypoid tumor within the submucosa and commonly occurs in the first part of the duodenum (Kloppel et al., 2004, 2007). MEN1-associated gastrin-producing NETs are multiple and tiny (sometimes less than 1 mm in diameter) (Anlauf et al., 2005; Kloppel et al., 2007). Histologically, gastrin-producing NETs show trabecular and pseudoglandular patterns with immunohistochemical gastrin expression (Figures 6A–D). The most common region in which somatostatin-producing NETs arise is the papilla of Vater (Figure 7A) (Burke et al., 1997; Garbrecht et al., 2008). About 43% of somatostatin-producing NETs and 14% of non-MEN1-associated somatostatin-producing NETs are associated with neurofibromatosis type 1 (Soga and Yakuwa, 1999; Garbrecht et al., 2008). Although somatostatin-producing NETs show immunohistochemical positivity for somatostatin (Figure 7D), somatostatin syndrome does not usually develop (Garbrecht et al., 2008). Somatostatin-producing NETs are arranged in a trabecular pattern with pseudoglandular structures (Figures 7B and C). Psammoma bodies are occasionally present (Figure 7C) (Soga and Yakuwa, 1999; Garbrecht et al., 2008). MEN1-associated somatostatin-producing NETs accompany somatostatin cell hyperplasia of the non-neoplastic mucosa (Garbrecht et al., 2008). Duodenal NECs most commonly arise in the papilla of Vater (Zamboni et al., 1990; Nassar et al., 2005; Kloppel et al., 2007). Nassar et al. reported 14 cases of ampullary NECs comprising 8 cases (57%) of large-cell neuroendocrine carcinoma and 6 cases (43%) of small-cell carcinoma (Nassar et al., 2005).

### Distal jejunum and ileum

Most distal jejunal and ileal NETs are serotonin-producing EC-cell NETs (Levy and Sobin, 2007). Carcinoid syndrome usually occurs due to the hormonal effects of serotonin when the tumor metastasizes to the liver (Kloppel et al., 2007; Levy and Sobin, 2007). Macroscopically, most NETs of the distal jejunum and ileum occur at the terminal ileum either as single small sessile nodules usually measuring between 1 and 2 cm or as multiple tumors (Burke et al., 1997; Yantiss et al., 2003; Kloppel et al., 2007). Histologically, EC-cell NETs proliferate in an insular growth pattern, with solid to cribriform tumor structures. Glandular-like structures or palisading of the peripheral cell layers are occasionally seen (Kloppel et al., 2004, 2007; Levy and Sobin, 2007). S-100 protein positivity has been reported in sustentacular-like cells in 7% of tumors (Burke et al., 1997).

### Appendix

**Figure 8:**
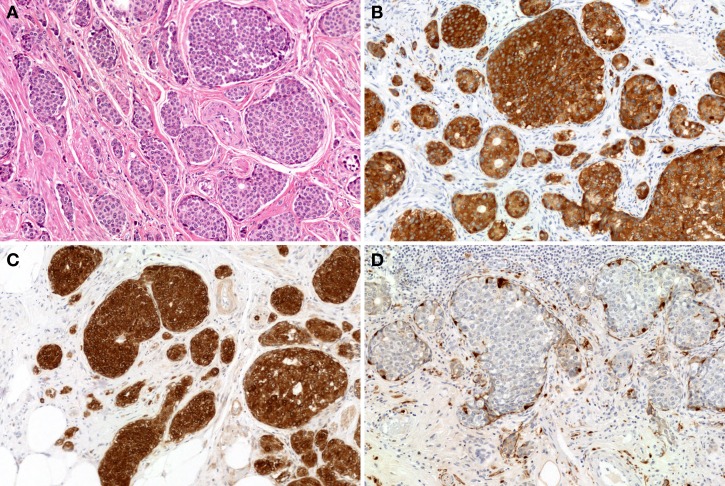
**EC-cell NETs of the appendix.** The tumor proliferates in an insular growth pattern, with solid to cribriform tumor structures (H and E stain, **A**). The tumor cells are positive for synaptophysin **(B)** and serotonin **(C)**. S-100 protein positivity is observed in sustentacular-like cells **(D)**.

**Figure 9:**
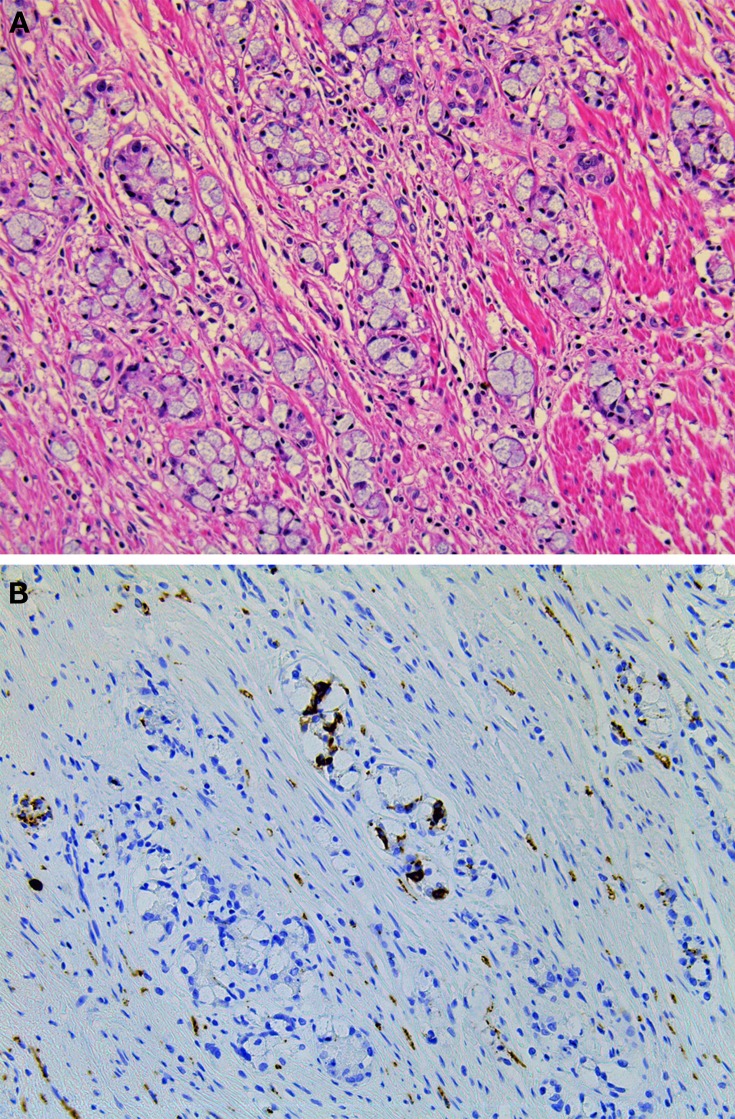
**Goblet cell carcinoid of the appendix. (A)** Tumor cells of goblet cell carcinomas possess intracytoplasmic mucus with small, compressed nuclei. **(B)** Tumor cells are focally positive for synaptophysin.

The most common site in which appendiceal NETs arise is the tip of the appendix (Carr and Sobin, 2004). EC-cell NETs account for a substantial portion of the appendiceal NETs (Carr and Sobin, 2004). The histological features of appendiceal EC-cell NETs are comparable to those of the ileal EC-cell NETs (Figures 8A–C) (Kloppel et al., 2004). S100-positive sustentacular cells have also been identified (Figure 8D) (Carr and Sobin, 2004). Two special types of appendiceal NENs are tubular carcinoid (TC) and goblet cell carcinoid (GCC). According to the current WHO classification, TC is classified as a NET, and GCC, as a MANEC (Bosman et al., 2010). TC forms discrete tubules and short lines of cells within an abundant stroma, and some contain mucus in the lumen (Carr and Sobin, 2004). Misdiagnosis TC as metastatic adenocarcinoma is a well-known pitfall about NET (Bosman et al., 2010). GCC is a unique histologic variation of NEN, showing both neuroendocrine and ductal differentiations (Roy and Chetty, 2010; Yong et al., 2010). GCC commonly proliferates in the lamina propria or submucosa at the tip of the appendix and macroscopically presents as a solid, hard, grayish ill-demarcated tumor (Roy and Chetty, 2010; Yong et al., 2010). Tumor cells of GCC possess intracytoplasmic mucus, which is similar to goblet cells or signet-ring cells, with small, compressed nuclei (Figure 9A) (Yong et al., 2010). Tumors proliferate with tight solid nests or tubules that have small lumina and invade the muscular layer, serosa, and mesoappendiceal tissues (Yong et al., 2010). GCCs are more aggressive than conventional NETs but less than adenocarcinomas of the appendix (Bosman et al., 2010). The Ki-67 index is >2% in 41% (7/17 cases) of GCC (Alsaad et al., 2007). Immunohistochemically, GCCs show strong carcinoembryonic antigen and cytokeratin positivity and are inconsistently positive for neuroendocrine markers such as chromogranin A and synaptophysin (Figure 9B) (Alsaad et al., 2007; Yong et al., 2010). GCCs have been reported to show CK20 positivity in 100% of cases (17/17 cases) and CK7 positivity in 71% (12/17 cases), whereas conventional NETs showed CK20 positivity in 16% of cases (4/25 cases) and CK7 negativity in all cases (Alsaad et al., 2007). Tang et al. showed that GCCs display a spectrum of histologic features and possess the potential to transform into adenocarcinomas, with either the signet-ring cell phenotype or the poorly differentiated adenocarcinoma phenotype. The adenocarcinoma ex GCC showed the worse prognosis (stage IV-matched 5-year survival: typical GCC, 100%; signet-ring cell type, 38%; poorly differentiated adenocarcinoma type, 0%) (Tang et al., 2008).

### Colon and rectum

**Figure 10:**
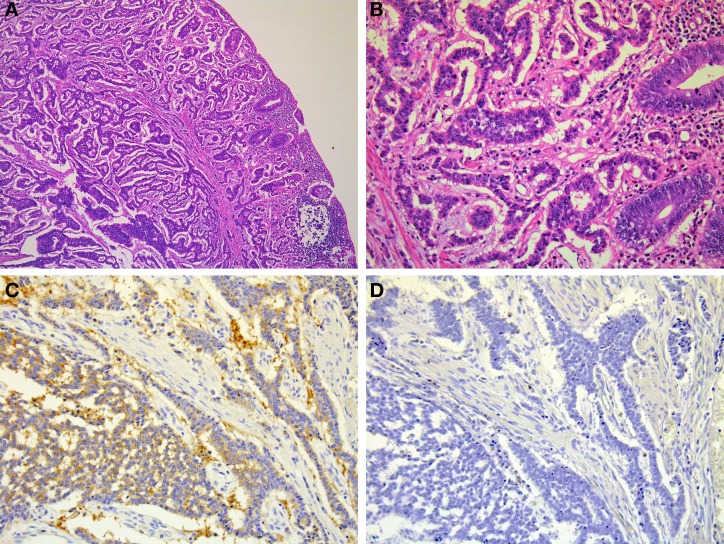
**Microscopic findings of rectal NET.** Rectal NET shows a trabecular proliferating pattern (**A**: low power view, **B**: high power view). Immunohistochemically, tumor cells of rectal NET are positive for synaptophysin **(C)** but negative for chromogranin A **(D)**.

Rectal NETs are more common than colonic NETs (Kloppel et al., 2007). Macroscopically, rectal NETs present as solitary sessile or semi-pedunculated tumors with intact overlying epithelium (Shim et al., 2004) (Figure 1). Larger neoplasms can be ulcerated (Shim et al., 2004). Histologically, rectal NETs show a characteristic trabecular pattern (Figures 10A and B) (Kloppel et al., 2007). Immunohistochemically, rectal NETs are usually positive for prostatic acid phosphatase and synaptophysin and negative for chromogranin A (Figures 10C and D) (Federspiel et al., 1990; Kloppel et al., 2007). The majority of colonic NETs are detected in the cecum (Soga, 1998). Histologically, colonic NETs proliferate with a nodular, trabecular, or mixed pattern (Soga, 1998; Kloppel et al., 2007). Immunohistochemically, NETs of the cecal region are positive for serotonin (Kloppel et al., 2007). NECs are more common in the colon, especially the right colon, than in the rectum (Kloppel et al., 2007; Bosman et al., 2010). Large-cell carcinoma is the most common colorectal NEC (51%, 19/37 cases), followed by small-cell carcinoma (24%, 9/37 cases) and mixed carcinoma of small cell carcinoma and large call carcinoma (24%, 9/37) (Shia et al., 2008). Colonic NECs are frequently associated with an overlying adenoma or adenocarcinoma but are not associated with NETs (Shia et al., 2008).

## Conclusions

Although GI-NENs have common histological features, microscopic and immunophenotypic features have to be considered in the different anatomical sites.

The recent WHO classification and ENETS grading systems are based on the Ki-67 index and mitotic count in histological material. Intratumoral heterogeneity, especially in biopsy and cytological materials, limits the diagnostic accuracy of the grading system. For accurate grading and pathological diagnosis, it is important to carefully evaluate hot spots for the Ki-67 index, identify areas of highest mitotic density for mitotic count, and recognize the characteristic histological features of GI-NENs.

### Conflict of interest statement

The authors declare that the research was conducted in the absence of any commercial or financial relationships that could be construed as a potential conflict of interest.
